# P-1916. Donor Dilemma: A Pilot Study of a Novel Curriculum to Address Gaps in Transplant ID Training

**DOI:** 10.1093/ofid/ofaf695.2085

**Published:** 2026-01-11

**Authors:** Robert S Tyler, Divisha Sharma, Drew W Charles, Rachel Burgoon, Yosra Alkabab, Scott R Curry, Courtney E Harris

**Affiliations:** Medical University of South Carolina, Johns Island, SC; Medical University of South Carolina, Johns Island, SC; Medical University of South Carolina, Johns Island, SC; Medical University of South Carolina, Johns Island, SC; MUSC, Charleston, South Carolina; Medical University of South Carolina, Johns Island, SC; Medical University of South Carolina, Johns Island, SC

## Abstract

**Background:**

Solid organ transplantation is a rapidly growing field within Infectious Diseases (ID), yet most ID trainees receive limited, non-standardized training in assessing donor suitability, particularly for deceased donors. Prior simulation-based approaches, such as the donor call simulation by Sigler et al., have shown effectiveness but are resource-intensive. To address this gap, we developed novel, sustainable, online case-based modules focused on transplant donor risk assessment and decision-making. The modules present real-world scenarios and conclude with teaching points and clinical pearls. This pilot study aims to evaluate the perceived usefulness and educational value of these modules in enhancing clinical reasoning skills among ID trainees.

**Image 1:**
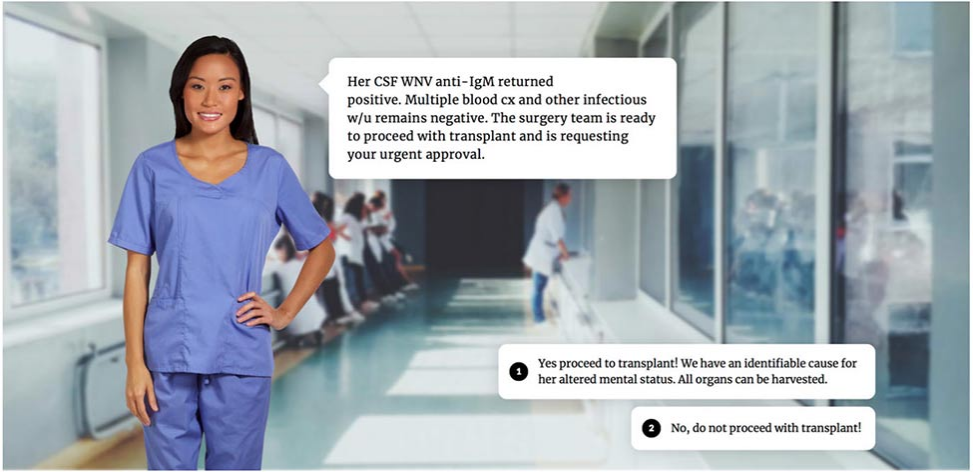
Example Interactive Case from ‘Donor with Unexplained Mental Altered Mental Status.’ In each case, learners are prompted with a case scenario and are tasked with obtaining additional information to guide management. In this case, learners are to recognize that West Nile Virus (WNV) encephalitis in the donor is an absolute contraindication to organ donation. Image Source: Articulate 360.

**Table 1:**
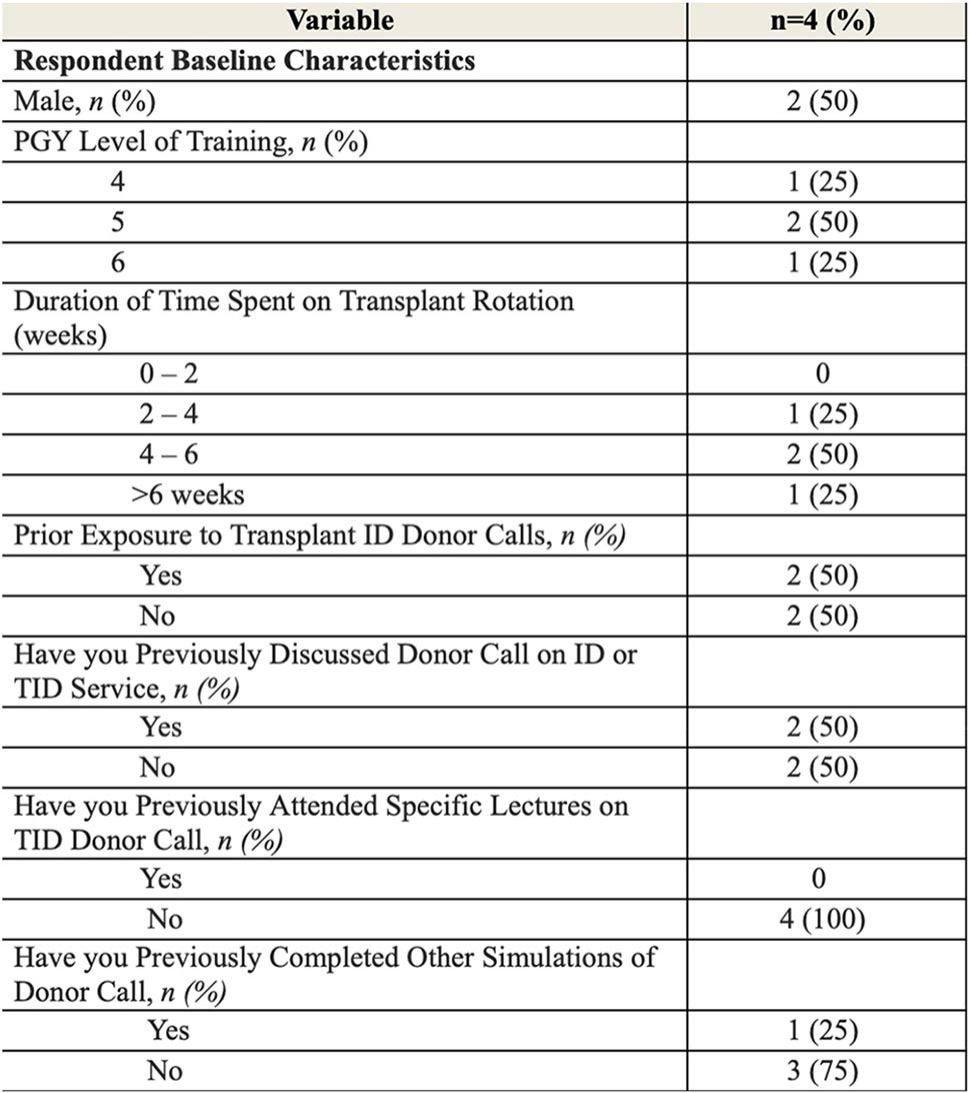
Respondent Baseline Characteristics

**Methods:**

Four ID fellows at our institution were assigned twelve case-based modules. Following completion of the modules, trainees completed a survey assessing perceived usefulness and benefit, knowledge gained, confidence in donor risk assessment, and likelihood of recommending the modules to other ID fellows.

**Table 2:**
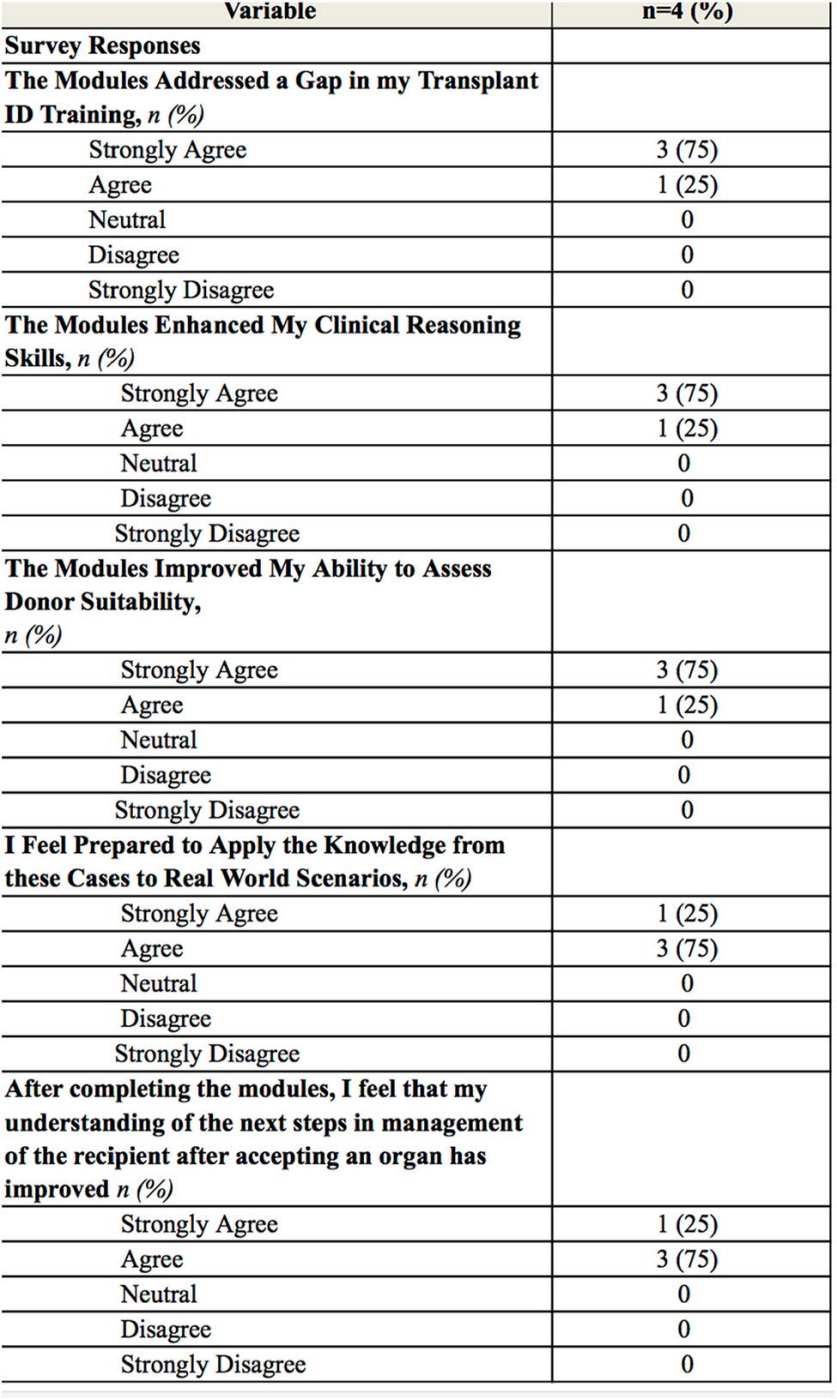
Survey Responses

**Results:**

Survey respondents included one first-year fellow, two second-year fellows, and one Transplant ID fellow. The median duration of prior experience on a transplant rotation was seven weeks. Three out of four respondents strongly agreed that the modules addressed a critical gap in their Transplant ID training. All participants found the modules to be both sustainable and practical as educational tools and unanimously reported that the modules improved their clinical reasoning skills, particularly in assessing donor organ suitability.

**Conclusion:**

This novel curriculum is the first sustainable training tool in Transplant ID specifically focused on donor risk assessment. All respondents agreed it filled a critical gap in their knowledge, enhanced their clinical reasoning, made them feel more prepared to assess donor suitability, and all would recommend them to their peers. This training model offers a scalable, high-impact approach to standardizing Transplant ID education and improving real-world clinical decision-making. Future research is planned to disseminate these modules nationally to assess their efficacy, continue to evaluate their impact, and grow this curriculum further.

**Disclosures:**

Rachel Burgoon, Pharm.D., Merck: Grant/Research Support

